# Rickettsioses in South Korea, Materials and Methods

**DOI:** 10.3201/eid1203.050334

**Published:** 2006-03

**Authors:** Pierre-Edouard Fournier, Jean-Marc Rolain, Didier Raoult

**Affiliations:** * Université de la Méditerranée, Marseille, France

**Keywords:** Rickettsia, rickettsiosis, serum, PCR

**To the Editor:** We read with interest the article by Choi et al. (*1*), which describes the molecular detection of *Rickettsia typhi* and 4 spotted fever group rickettsiae by nested polymerase chain reaction (PCR) in the serum of febrile Korean patients. The value of the study, however, is limited by imprecision, inconsistencies, and the impossibility of verifying data. First, neither epidemiologic nor clinical data are provided for studied patients, although these are essential for interpreting PCR results. Second, multiplex nested PCR is hampered by a high risk of contamination (*2*). Alternatively, nested PCR techniques that use a closed assay or single-use primers without positive controls limit such a risk (*3*). In all cases, the use of negative controls is critical (*2**,**3*). In this study, negative controls are neither described in the Materials and Methods section nor shown on the gels. In addition, the authors used as positive controls 4 of the 5 *Rickettsia* species they detected. Therefore, apart from *R. felis*, which was not used as a positive control, positive products may result from cross-contamination. Finally, technically, the data are impossible to reproduce: 1) primer sets WJ77/80 and WJ79/83/78 cited in the legends of Figures 2 and 3 are neither described nor referenced in the text, 2) sequence of the RpCS.877p primer in Table 1 differs from that in the referenced article (*4*), 3) described sequences have not been deposited in GenBank, and 4) all *rompB* primers described in Table 1 exhibit 1–6 nucleotide mismatches with *ompB* sequences of at least 1 of the detected species. Based on these errors, the 7 cases of dual infections with *R. conorii* and *R. typhi*, which have never been reported before, are doubtful, and these data need to be confirmed.

## References

[1] Choi YJ, Jang WJ, Kim JH, Ryu JS, Lee SH, Park KH, Spotted fever group and typhus group rickettsioses in humans, South Korea. Emerg Infect Dis. 2005;11:237–44. 15752441 10.3201/eid1102.040603 PMC3320442

[2] Hayden RT. In vitro nucleic acid amplification techniques. In: Persing DH, Tenover FC, Versalovic J, Tang YW, Unger ER, Relman DA, et al., editors. Molecular microbiology. Washington: ASM Press; 2003. p. 43–69.

[3] Fournier PE, Raoult D. Suicide PCR on skin biopsy specimens for diagnosis of rickettsioses. J Clin Microbiol. 2004;42:3428–34. 10.1128/JCM.42.8.3428-3434.2004 15297478 PMC497613

[4] Roux V, Rydkina E, Eremeeva M, Raoult D. Citrate synthase gene comparison, a new tool for phylogenetic analysis, and its application for the rickettsiae. Int J Syst Bacteriol. 1997;47:252–61. 10.1099/00207713-47-2-252 9103608

